# Noise Reduction Combining a General Microphone and a Throat Microphone

**DOI:** 10.3390/s22124473

**Published:** 2022-06-13

**Authors:** Junki Kawaguchi, Mitsuharu Matsumoto

**Affiliations:** Graduate School of Informatics and Engineering, University of Electro-Communications, 1-5-1, Chofugaoka, Chofu-shi 182-8585, Japan; k2230046@edu.cc.uec.ac.jp

**Keywords:** noise reduction, throat microphone, sensor fusion

## Abstract

In this study, we propose a method to reduce noise from speech obtained from a general microphone using the information of a throat microphone. A throat microphone records a sound by detecting the vibration of the skin surface near the throat directly. Therefore, throat microphones are less prone to noise than ordinary microphones. However, as the acoustic characteristics of the throat microphone differ from those of ordinary microphones, its sound quality degrades. To solve this problem, this study aims to improve the speech quality while suppressing the noise of a general microphone by using the information recorded by a throat microphone as reference information to extract the speech signal in general microphones. In this paper, the framework of the proposed method is formulated, and several experiments are conducted to evaluate the noise suppression and speech quality improvement effects of the proposed method.

## 1. Introduction

In recent years, with the development of information and communication devices, there has been an increase in the use of devices that use voice input, such as mobile devices, e.g., smartphones, for making calls. Although the use of voice input devices is comfortable in a noise-free environment, they may be used in a noisy environment. In such a case, the voice may be difficult to hear or unbearable to use. Therefore, speech enhancement is one of the important issues in acoustic processing to emphasize the target speech from the mixed signal.

The approach for speech enhancement can be categorized into two types. One is the approach using a single-channel signal. The other is the approach using multi-channel signals. The survey on speech enhancement classifies algorithms for speech enhancement of single-channel signals into three categories [1]. 

The spectral subtraction (SS) algorithm is one of those approaches. Early approaches include spectral subtraction in the correlation domain proposed by Weiss et al. [2] and that in the Fourier transform domain proposed by Boll [3]. Spectral subtraction is a powerful approach to single-channel speech enhancement and has undergone various improvements [4]. Another approach is a statistical model-based algorithm. For example, McAulay et al. used the maximum likelihood method to estimate the Fourier transform spectrum of the target signal [5]. The other approach is a subspace algorithm. Dendrinos et al. proposed an algorithm that uses singular value decomposition [6]. Ephraim et al. proposed an algorithm that uses an eigenvalue decomposition of signals [7]. The single-channel signal approach is attractive in that it uses only a single recording source. However, information that can be used for speech enhancement is limited to that related to the sound source itself. In recent years, deep learning-based approaches have also been actively studied but require a lot of computational power [8,9,10].

The microphone array is a typical method using multi-channel signals [11,12]. A microphone array uses multiple microphones to achieve noise reduction using the phase and amplitude differences of the sound entering each microphone. The microphone array is implemented in various ways. However, in principle, its implementation requires special equipment, such as a large number of microphones and complex processing. There have been various approaches to the study of noise reduction techniques. In general, noise reduction techniques using microphones have been widely studied. Well-known examples are sound focusing by phase homomorphism and adaptive microphone array by generating the zero point in the noise direction. In the field of blind source separation, independent component analysis [13,14,15] and sparsity-based source separation [16,17] are well known, but they require special equipment, such as a large number of microphones, for implementation.

One way to use voice input devices comfortably without being affected by noise is to use a microphone that is less susceptible to noise. A throat microphone is an example and records sound by detecting the vibration of the skin near the throat directly. Compared to general microphones that use air vibration to record, the throat microphone is airless and, therefore, more resistant to noise. Some studies paid attention to the characteristics and aimed to improve the accuracy of speech recognition by using a throat microphone that records by directly detecting the vibration of the skin near the throat [18]. Although the feature of throat microphones is useful, as described above, their acoustic characteristics differ from those of ordinary microphones, resulting in degraded speech quality. 

In image processing fields, there are some studies on sensor fusion to obtain better image quality. Cross bilateral filter (Joint bilateral filter) is a filter technique that combines non-flash images with flash images taken at the same location [19,20]. The filter inputs are flash and non-flash images, resulting in a more natural quality image as the output. Other research cases have been reported that combine infrared and visible camera images to detect various objects, such as faces [21], pedestrians [22] and cars [23]. 

Inspired by these studies, we apply these concepts to the acoustical field and aim to obtain more natural speech by eliminating noise in speech signals obtained by a general microphone by using speech signals obtained by a throat microphone as a reference. 

In this study, we propose a noise reduction method for general microphones based on speech signals from throat microphones. We also evaluate the effectiveness of the proposed method by conducting experiments. The rest of the paper is summarized as follows: In Section 2, we describe the typical binary mask using two microphones to clarify the proposed method. In Section 3, we describe the proposed approach and the usage scenario. In Section 4, some experiments are conducted using the proposed method to evaluate the noise reduction performance of the proposed method and the sound quality compared to a throat microphone. In Section 5, we present a discussion and conclusions on the results of the experiment and future perspectives.

## 2. Binary Mask Using Two Microphones

### 2.1. Problem Formulation

To clarify our approach, we first show the typical method using the binary mask with two general microphones [24,25,26]. A typical approach using binary masks assumes two microphones. For the mixed signal *x*_1_(*t*) obtained from microphone 1, removing the effects of attenuation and delay does not lose the generality of the problem. Hence, *x*_1_(*t*) can be written as follows:

(1) $$x_{1}\left(t\right)=s\left(t\right)+\sum_{i=1}^{N}n_{i}\left(t\right),$$

where *s*(*t*) is the objective signal, and *n_i_*(*t*) is the *i*th noise (*i* = 1, 2, 3, …, *N*). On the other hand, it is necessary to consider the delay and attenuation of microphone 2’s signal with respect to the signal acquired by microphone 1. Therefore, the second mixed signal *x*_2_(*t*) can be written as:

(2) $$x_{2}\left(t\right)=as\left(t-\delta \right)+\sum_{i=1}^{N}a_{i}n_{i}\left(t-\delta_{i}\right),$$

where *δ* and *δ_i_* are the delays from the objective signal and the delay from the noise *j*, respectively. *a* and *a_j_* are parameters that indicate the relative attenuation between the sensors for the objective signal and noise, respectively.

When we define Δ as the maximum possible delay between sensors, the following constraints are obtained:

(3) |δ|≤Δ,

(4) $$\left|\delta_{i}\right|\leq \Delta ,$$

The sparse approach assumes that the target signal and noise are sparse in the time-frequency domain. Let *S*(*τ*, *ω*) and *N_i_*(*τ*, *ω*) be the short-time Fourier transform of *s*(*t*) and *n_j_*(*t*), respectively. *S*(*τ*, *ω*) can be described as follows:

(5) $$S\left(\tau ,\omega \right)=\sum_{\tau =-\infty }^{\infty }s\left(t+\tau \right)W\left(\tau \right)\exp\left(-i\omega \tau \right),$$

where *τ* and *ω* are the time frame and the angular frequency, respectively. *W*(*τ*) is the window function. If the sparsity between the objective signal and all the noise is satisfied, we can obtain the following condition:

(6) $$S\left(\tau ,\omega \right)N_{i}\left(\tau ,\omega \right)=0\;\forall \tau ,\omega ,$$

### 2.2. Noise Reduction Using Binary Mask

In order to perform denoising, the delay and attenuation parameters in Equation (2) are estimated. If *X_i_*(*τ*, *ω*) is a short-time Fourier transform of *x_i_*(*t*), it can be written as follows.

(7) $$X_{i}\left(\tau ,\omega \right)=\sum_{t=-\infty }^{\infty }x_{i}\left(t+\tau \right)W\left(t\right)\exp\left(-i\omega t\right),$$

The signals obtained by microphones 1 and 2 can be rewritten in the time-frequency domain as follows.

(8) $$\left[\begin{array}{c} X_{1}\left(\tau ,\omega \right)\\ X_{2}\left(\tau ,\omega \right) \end{array}\right]=\left[\begin{array}{cccc} 1 & 1 & \ldots & 1\\ a\exp\left(-i\omega \delta \right) & a_{1}\exp\left(-i\omega \delta_{1}\right) & \ldots & a_{N}\exp\left(-i\omega \delta_{N}\right) \end{array}\right]\left[\begin{array}{c} S\left(\tau ,\omega \right)\\ N_{1}\left(\tau ,\omega \right)\\ \begin{array}{c} \ldots \\ N_{N}\left(\tau ,\omega \right) \end{array} \end{array}\right]$$

If the target signal and noise are sparse in the time-frequency domain, the following equation is obtained:

(9) $$\left[\begin{array}{c} X_{1}\left(\tau ,\omega \right)\\ X_{2}\left(\tau ,\omega \right) \end{array}\right]=\left[\begin{array}{c} 1\\ a\left(\tau_{s},\omega_{s}\right)\exp\left(-i\omega_{s}\delta \left(\tau_{s},\omega_{s}\right)\right) \end{array}\right]s\left(\tau_{s},\omega_{s}\right)$$

where the objective signal exists at (*τ_s_*, *ω_s_*). To estimate (*τ_s_*, *ω_s_*), we calculate the ratio of *X*_1_(*τ_s_*, *ω_s_*) and *X*_2_(*τ_s_*, *ω_s_*). Let us define *a*(*τ_s_*, ω_s_) and *δ*(*τ_s_*, *ω_s_*) as the relative amplitude and delay parameters. We can estimate *a*(*τ_s_*, *ω_s_*) and *δ*(*τ_s_*, *ω_s_*) as follows:

(10) $$\left(a\left(\tau_{s},\omega_{s}\right),\delta \left(\tau_{s},\omega_{s}\right)\right)=\left(\left|\frac{X_{2}\left(\tau ,\omega \right)}{X_{1}\left(\tau ,\omega \right)}\right|,\frac{1}{\omega }∠\frac{X_{2}\left(\tau ,\omega \right)}{X_{1}\left(\tau ,\omega \right)}\right)$$

where

(11) ∠aexp(iϕ)=ϕ, −π<ϕ<π,

We can design the time-frequency mask *M*(*τ*, *ω*) for (*τ*, *ω*) as follows:

(12) $$M\left(\tau ,\omega \right)=\{\begin{array}{c} 1,\left|\ln a\left(\tau ,\omega \right)-\ln a\right|<\frac{\Delta_{a}}{2}\;\wedge \left|\ln\delta \left(\tau ,\omega \right)-\ln\delta \right|<\frac{\Delta_{\delta }}{2}\\ 0,\;otherwise \end{array}$$

where Δ*_a_* and Δ*_δ_* are defined as the amplitude and delay resolution width of the histograms, respectively. In order to remove noise from the signal obtained by microphone 1, the masking process is executed as follows.

(13) $$S\left(\tau ,\omega \right)=M\left(\tau ,\omega \right)X_{1}\left(\tau ,\omega \right),$$

Although the noise reduction performance of the binary mask is high, its quality depends on the accuracy of the estimated masking information. In the presence of environmental noise, the noise does not fully meet the sparsity assumptions, making it difficult to estimate mask information.

## 3. Proposed Approach

### 3.1. Overview of the Proposed Method

**Figure 1:**
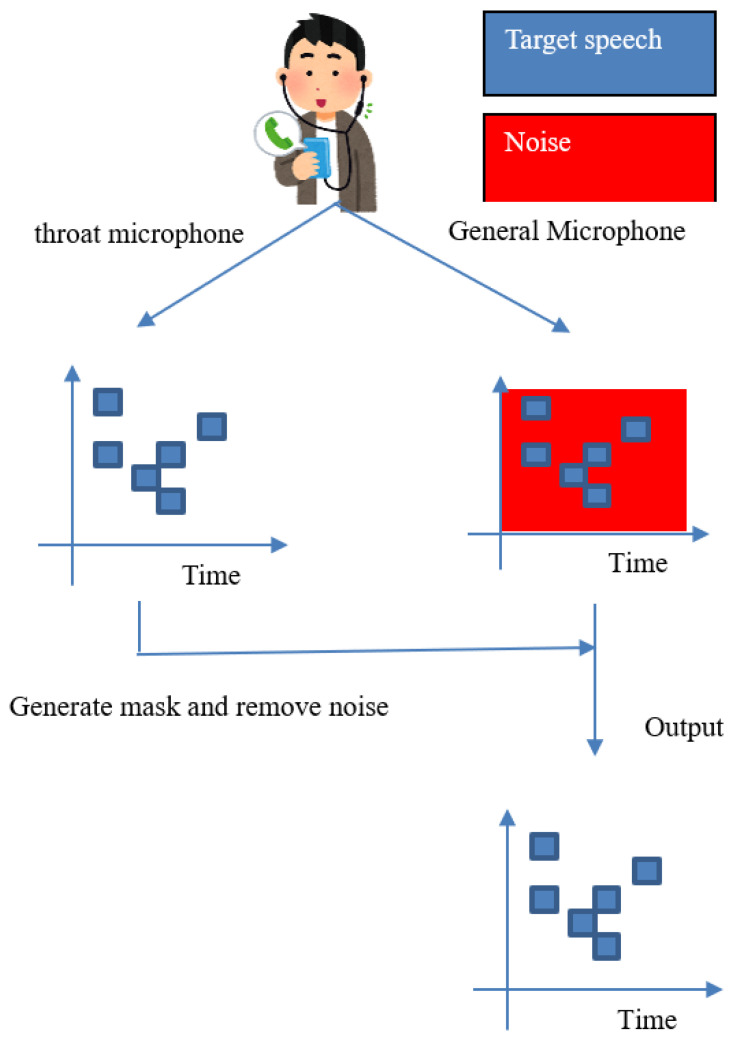
Overview of the proposed method.

Figure 1 shows an overview of the proposed method. In our supposed scenario, we record the sound with a general microphone and a throat microphone simultaneously. We then create a binary mask based on the speech data obtained from the throat microphone to remove noise from the speech data obtained from the general microphone.

To achieve this goal, speech data is first captured by both a throat microphone and a general microphone. We then transformed the sound into frequency domain data by Fourier transform. The next step is to set a threshold value for the power spectrum of the speech data from the throat microphone and create a binary mask with 1 for the power above the threshold value and 0 for the rest. The audio data from the general microphone is also subjected to Fourier transform and converted to frequency domain data. The binary mask is applied to the obtained frequency data from the general microphone to remove noise. Finally, the noise-removed signal is inverse Fourier transformed to obtain the output signal.

### 3.2. Formulation by Equation

We formulate the proposed approach in this section. Let *x*_1_(*t*) be the mixed signal obtained from a general microphone and *x*_2_(*t*) be the signal obtained from a throat microphone. *T* is time. The spectra of *x*_1_(*t*) and *x*_2_(*t*) are denoted as *X*_1_(*τ*, *ω*) and *X*_2_(*τ*, *ω*), respectively, when the signals are mapped into frequency space by Fourier transform. *τ* is the time frame, and *ω* is the angular frequency. When the spectrum of the objective signal is *S*(*τ*, *ω*) and the spectrum of the *i*th noise is *N_i_*(*τ*, *ω*), *X*_1_(*τ*, *ω*) is expressed as follows.

(14) $$X_{1}\left(\tau ,\omega \right)=S\left(\tau ,\omega \right)+\sum_{i=1}^{N}N_{i}\left(\tau ,\omega \right),$$

On the other hand, the signal of the throat microphone does not contain noise signals, while the frequency response of the sound recorded by the throat microphone is different from that of the microphone. *X*_2_(*τ*, *ω*) is expressed as follows.

(15) $$X_{2}\left(\tau ,\omega \right)=B\left(\omega \right)S\left(\tau ,\omega \right),$$

where *B*(*ω*) is the frequency response of a throat microphone to a general microphone. For noise reduction, a threshold is set, and a binary mask *M*(*τ*, *ω*) is generated as follows. *th* is a threshold.

(16) $$M\left(\tau ,\omega \right)=\{\begin{array}{c} 1\;\;\;\;\;\;\;\;\;|X_{2}\left(\tau ,\omega \right)|\geq th\;\\ 0\;\;\;\;\;\;\;\;\;\left|X_{2}\left(\tau ,\omega \right)\right|<th\; \end{array}$$

Based on the mask information, the following process is applied to the spectral signal *X_1_*(*τ*, *ω*) of a general microphone to remove noise and obtain the output signal spectrum *Y*(*τ*, *ω*).

(17) $$Y\left(\tau ,\omega \right)=M\left(\tau ,\omega \right)X_{1}\left(\tau ,\omega \right)$$

Finally, the obtained signal *Y*(*τ*, *ω*) is inverse Fourier transformed to obtain the output signal *y*(*t*). Binary mask estimation using the microphone ratio is difficult if sparse assumptions are not met. 

On the other hand, our method obtains mask information directly from the throat microphone. Therefore, the proposed method can realize efficient noise removal even for a voice that does not satisfy the assumption of sparsity.

We also note that the delay between the throat microphone and the general microphone is not taken into account. The main reason is due to the difference between the typical binary mask usage scenario and the proposed method usage scenario. In the typical binary mask usage scenario, the speaker is apart from the listener, and the listener records the sound from the speaker. The proposed method aims to record the speaker’s voice clearly in a situation where the surroundings are extremely noisy. Since this work is performed on the speaker side, it is assumed that recording with a microphone and a throat microphone will be performed at the same time.

## 4. Experiment

### 4.1. Experimental Method

The effectiveness of the method proposed in Section 2 is evaluated by experiments. In this study, the accuracy of the noise removal is verified by creating the target speech, noise speech and mixed speech in advance. For the noise, we used sound effects from the Sound Effects Lab [27] and Hashimoto Research Institute [28]. Recorded speech was used as the voice. Recording with the general microphone and throat microphone was performed at the same time in a normal quiet room. The used general microphone was M4U made by inMusic, Inc. (Cumberland, RI, USA). The used throat microphone was DN-915129 made by ThirdWave Co., Ltd. (Tokyo, Japan). To evaluate the noise reduction performance quantitatively, the mixtures were created on a computer. All programs were written in Python. 

The experimental conditions are shown in Table 1. Three types of noise signals were synthesized for each target signal, for a total of three types of signals. The noise level was described by dBFS. The threshold was changed in 10 dB steps for noise removal. A Hamming window was used as the window function. In this study, Signal to Distortion Ratio (SDR) [29], Signal to Noise Ratio (SNR) and Signal to Interference Ratio (SIR) [24] were used for evaluation between the target speech and the denoised speech.

The Signal to Distortion Ratio (SDR) is a measure of how much the speech is distorted relative to the target speech after noise reduction. *Ŝ*(*τ*, *ω*) is the signal to be evaluated for the target signal, and *S*(*τ*, *ω*) is the target signal. SDR is defined as follows.

(18) $$SDR=10\log_{10}\left(\frac{\sum_{\tau ,\omega }\left|S\left(\tau ,\omega \right)\right|}{\sum_{\tau ,\omega }\left|\left|S\left(\tau ,\omega \right)\right|-\lambda \left|\hat{S}\left(\tau ,\omega \right)\right|\right|\;}\right)$$

*λ* is a parameter used to normalize the power of the signal and is expressed as follows.

(19) $$\lambda =\sqrt{\frac{\sum_{\tau ,\omega }\left|S\left(\tau ,\omega \right)\right|}{\sum_{\tau ,\omega }\left|\hat{S}\left(\tau ,\omega \right)\right|}}$$

The signal-to-noise ratio (*SNR*) is the ratio of the power of the signal to that of the noise, which is used to evaluate how much noise is included in the signal mixture. The higher the value of *SNR* is, the smaller the effect of noise is. If *S*(*τ*, *ω*) is the original target signal and *N*(*τ*, *ω*) is the noise signal, *SNR* is defined as follows.

(20) $$SNR=10\log_{10}\left(\frac{\sum_{\tau ,\omega }\left|S\left(\tau ,\omega \right)\right|}{\sum_{\tau ,\omega }\left|N\left(\tau ,\omega \right)\right|\;}\right)$$

On the other hand, the signal to interference ratio (*SIR*) can be used to evaluate how much noise is contained in the output after applying the binary mask [11]. Assuming that the binary mask is *M*(*τ*, *ω*), *SIR* is defined as follows:

(21) $$SIR=10\log_{10}\left(\frac{\sum_{\tau ,\omega }\left|M\left(\tau ,\omega \right)S\left(\tau ,\omega \right)\right|}{\sum_{\tau ,\omega }\left|M\left(\tau ,\omega \right)N\left(\tau ,\omega \right)\right|\;}\right)$$

By comparing these results, it is possible to understand the effect of noise on the target signal of the speech after denoising. 

### 4.2. Quantitative Evaluation of Experiments

The SDR of the throat microphone to the signal obtained by a standard microphone was 0.474 dB in noise-free conditions. Table 2, Table 3 and Table 4 show the *SDR* values between the target signal and the speech after noise reduction and the *SIR* between the noise signal and the speech after noise reduction when three types of noise are mixed, respectively.

The experimental results are expressed in four significant digits. The background color is yellow when the *SDR* value is the highest. Table 2, Table 3 and Table 4 show that the *SDR* values of the noise-eliminated speech are larger than those of the throat microphone in all cases, indicating that the speech recorded by the noise-eliminated speech is closer to that recorded by a conventional microphone. Next, the *SNR*s of each noise signal and the target signal are shown in Table 5. 

Noise is unlikely to be mixed into the voice recorded by the throat microphone. However, the frequency response of the sound recorded by the throat microphone is different from that of the microphone. *SDR* shows the similarity to clear voice recorded with a general microphone. Hence, the fact that the *SDR* at the output of the proposed method is higher than that of the throat microphone means that the proposed method can be used to obtain a sound closer to the sound recorded by a normal microphone than by recording with a throat microphone. 

**Figure 2:**
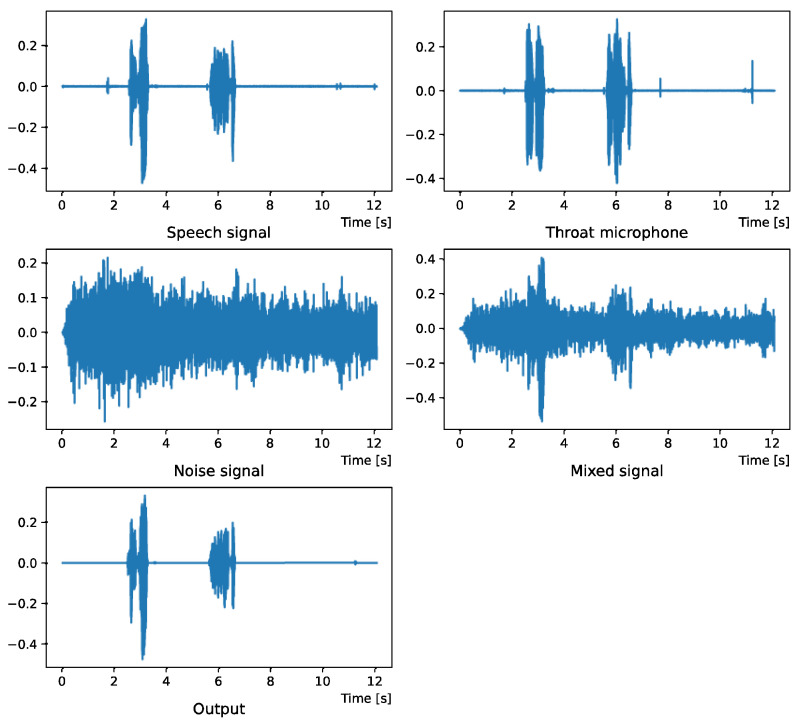
Waveform (Intersection noise).

**Figure 3:**
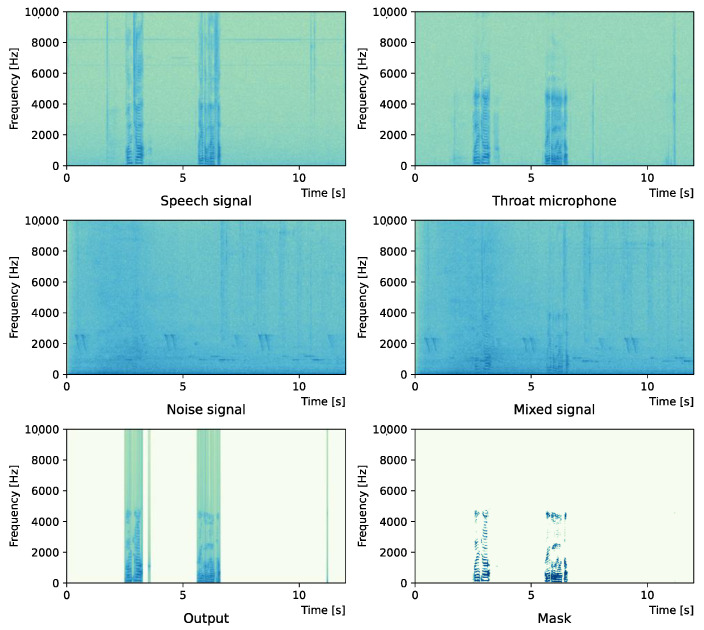
Spectrogram (Intersection noise).

**Figure 4:**
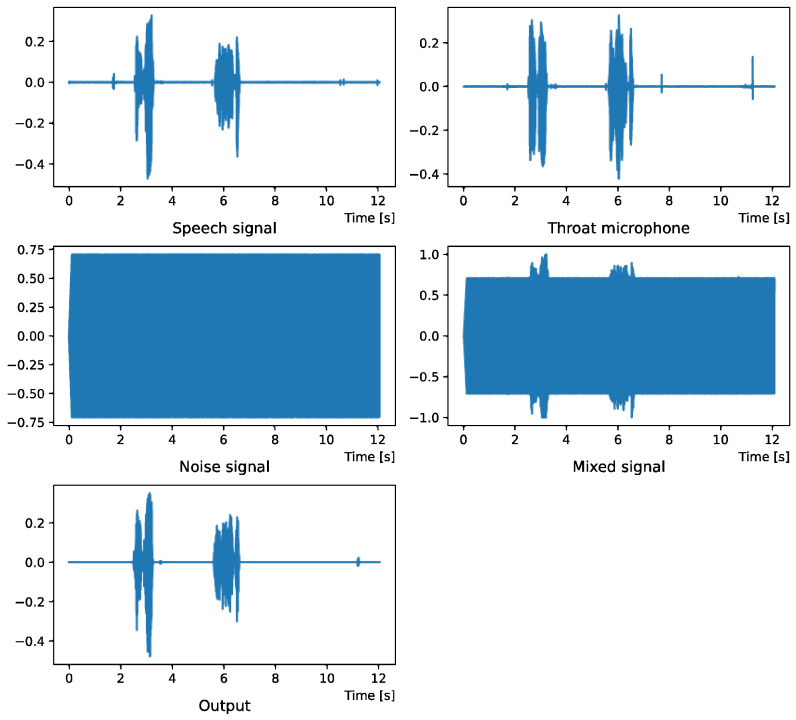
Waveform (White noise (0 dB)).

**Figure 5:**
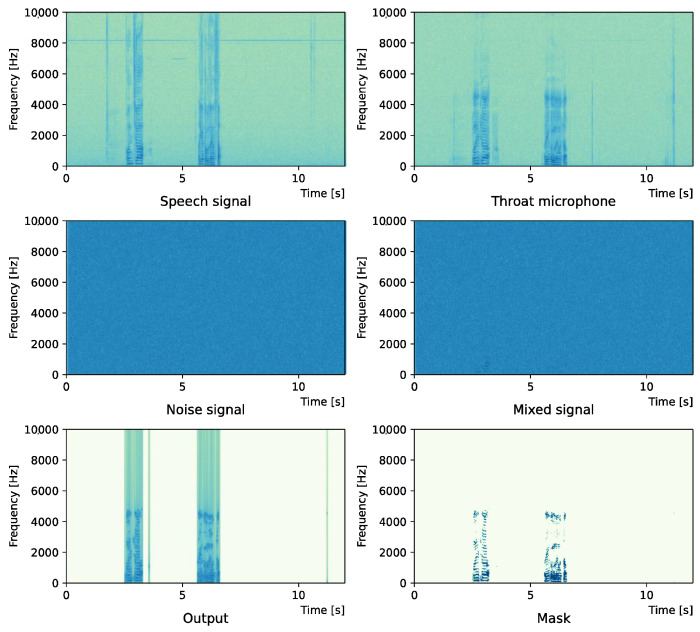
Spectrogram (White noise (0 dB)).

**Figure 6:**
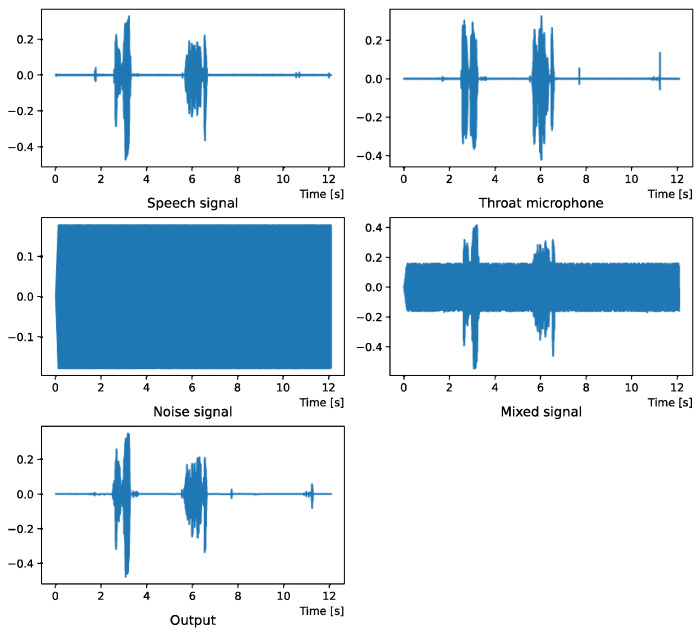
Waveform (White noise (−15 dB)).

**Figure 7:**
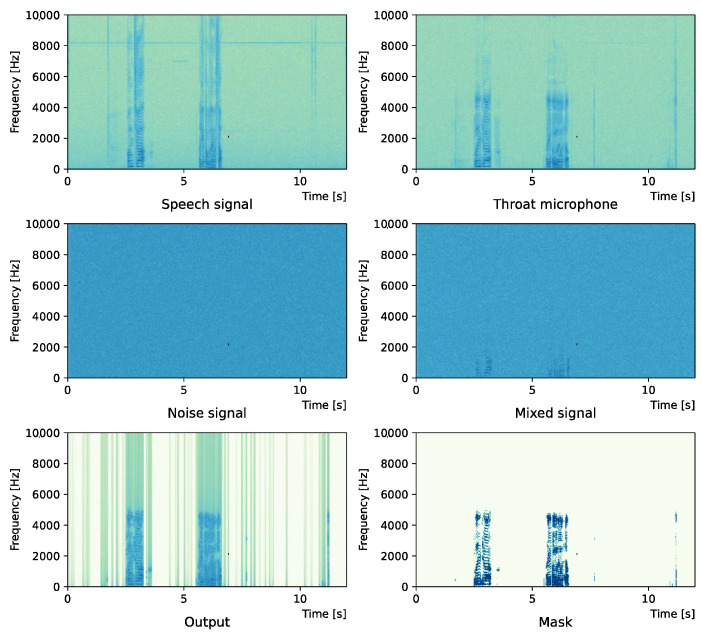
Spectrogram (White noise (−15 dB)).

Comparing the *SNR* values in Table 5 with the SIR values in Table 2, Table 3 and Table 4, it was confirmed that under the same noise conditions, the speech after denoising was larger, and the effect of noise was reduced. The waveform and spectrogram of the results obtained in the experiment are shown in Figure 2, Figure 3, Figure 4, Figure 5, Figure 6 and Figure 7. The spectrograms and waveforms for the maximum *SDR* among all the threshold values are shown in the figures. Figure 3, Figure 5 and Figure 7 also show the figure of the binary mask. It is noted that the output spectrogram is colored even in the high-frequency range, but the actual power is almost zero. This is due to the rounding error caused by outputting the spectrogram again after obtaining the output waveform once by the proposed method.

Figure 2 and Figure 3 show the waveform and the spectrogram, respectively, when intersection noise is used as noise.

Figure 4 and Figure 5 show the waveform when we used white noise whose noise level was 0 dB. Figure 6 and Figure 7 show the waveform when we used white noise whose noise level was −15 dB. As shown in Figure 2, Figure 3, Figure 4, Figure 5, Figure 6 and Figure 7, even if the signal is buried in noise, the objective signal can be extracted well.

## 5. Discussion and Conclusions

In this study, we proposed a noise reduction method using a combination of a throat microphone and a conventional microphone. In the proposed method, a throat microphone is used as a reference signal to remove the noise in the signal obtained from a general microphone.

In the experiments, white noise and general noise were used as mixtures, and SDR and SIR were used as evaluation criteria to evaluate the noise suppression performance and speech quality. The results showed that the proposed method was able to remove noise. The proposed method applies a mask created based on the information of the throat microphone to the sound recorded by a general microphone, and it is considered that noise is mixed in the remaining frequency range. However, since voice information is sparse in the frequency domain, it is considered that the SN ratio after masking is improved even if the SN ratio is bad in the time domain. As a result, it is considered that masked sound from the general microphone has better audio quality than the sound from the throat microphone. 

In the experiment, the voice of the speaker was recorded simultaneously using both a microphone and a throat microphone. Strictly speaking, there may be some time lag, but good results have been obtained experimentally without considering it.

However, there are some problems to be solved in the proposed method. For example, the frequency range in which a throat microphone can record is lower than the frequency range in which a general microphone can record. One solution is to apply the proposed method only to the frequency domain where the throat microphone can record. However, even with this approach, noise remains in the high-frequency components. The inability to record high-frequency components is a problem caused by the use of piezoelectric elements in the throat microphone. Hence, it is necessary to improve the sensor in order to substantially improve this problem.

In order to put this method to practical use, we are thinking of implementing the proposed method in real-time. We would like to proceed with experiments using the signals recorded in real noisy environments in the future. We also would like to investigate a method to estimate the threshold value automatically. 

## Figures and Tables

**Table 1:** Experimental condition.

Target Speech	Male Voice
Noise signal	Intersection noise, white noise (0, −15 [dB])
Noise reduction threshold	−90 to −30 [dB] (in 10 [dB] steps)

**Table 2:** Intersection noise.

Threshold (dB)	*SDR*	*SIR*
−90	0.704	−3.347
−80	2.632	0.008068
−70	8.384	7.795
−60	9.593	12.97
−50	7.393	15.50
−40	4.351	17.10
−30	1.527	20.83

**Table 3:** White noise (0 dB).

Threshold (dB)	*SDR*	*SIR*
−90	1.665	−9.767
−80	3.199	−7.361
−70	5.007	−4.756
−60	6.838	−1.369
−50	6.646	2.083
−40	4.236	5.033
−30	1.522	8.821

**Table 4:** White noise (−15 dB).

Threshold (dB)	*SDR*	*SIR*
−90	7.510	2.233
−80	9.579	4.639
−70	10.66	7.244
−60	9.764	10.63
−50	7.534	14.08
−40	4.415	17.03
−30	1.540	20.82

**Table 5:** SNR results.

Noise Speech	*SNR*
Intersection noise	−4.158
White noise (0 dB)	−22.96
White noise (−15 dB)	−10.96
